# On the Stations Nosologically Assigned to Synocha, or Inflammatory Fever, and to the West India Yellow Fever

**Published:** 1818-05

**Authors:** Nodes Dickinson

**Affiliations:** of the College of Surgeons


Medico-Chirurgical

JOURNAL AND REVIEW.

VOL. V.] MAY, 1818. [NO. 29.

### PART I.

### ORIGINAL COMMUNICATIONS.

——— quid utile ———

### *On the Stations nosologically assigned to Synocha, or inflammatory Fever, and to the West India Yellow Fever.*

By NODES DICKINSON, of the College of Surgeons.

THE symptom of increased heat has given to certain morbid affections the designation of *Fever,* literally appropriate; but not sufficiently discriminative of "Idiopathic," or, Fever "strictly so called," if it is made to comprehend diseases in which, although heat is a predominant appearance, yet other important characteristics of the Order *Febres,* or *Pyretica,* are wanting.

Thus, Synocha—general inflammation—inflammatory fever; a disease accurately marked by—"Heat greatly increased; pulse quick, hard, and strong; urine red; disturbance of the mind slight:"—"Produced by the stimulus of violent passions, undue muscular exercise, or heating foods, upon a plethoric habit; as also by a suppression of accustomed discharges, as those of menstruation, habitual venæsection, or perspiration"—is included, within the common designation of fever, as a species of the same genus with typhus.*

* *See* a Physiological System of Nosology, by John Mason Good, F. R S. &c. &c. &c.—from whence these "*distinctive*" characters of

The affinity, in this arrangement, cannot be considered to arise from any *distinctive* symptoms by which these diseases *identify* themselves. For, typhus is defined with "pulse small, weak, and unequal; usually frequent; heat and urine nearly natural; great prostration of strength, and disturbance of the mental powers." While the only circumstance stated in common to synocha and typhus, is "one series of increase and decrease; with a *tendency* to exacerbation and remission."

Nor does there appear a more strict analogy in the causes. Those productive of synocha have been adverted to above. The cause of typhus is usually regarded to be contagion.

With respect to the intermittent and remittent genera, they are commonly, if not always, derived from marsh miasmata; or, from a state of soil analogous thereto.

Whatever may be the essential nature of the effluvium of marshy lands, or of the *peculiar* morbid change it effects in the natural state of the living system; a disease is occasioned by its agency, singularly *characteristic* of its specific power: "Intermitting, and returning during (its) course"—otherwise "strikingly exacerbating and remitting."

If then, *idiopathic* fevers of type, and contagious continued fever arise from specific "morbid miasms" which, (however various or alike among themselves are yet undoubtedly dissimilar and distinct from the stimuli productive of synocha) excite the symptoms of their respective species through the medium of a *peculiar* change in the vital actions; and if this morbid change is characterized, in *both* cases, by "*extreme* debility"—"Pulse small, weak, and unequal;"—while the morbid affection which has been stationed with those forms of disease under the denomination of synocha, is produced by the stimulus of "violent passions, undue muscular exercise, &c.;" occasioning "heat greatly increased; pulse quick, hard and strong."—Without adverting, at present, to the distinctly different principle which must direct their treatment, I beg to inquire with what hope of practical ad-

---

fever have been taken. The features delineated by the learned author of that (in many respects) enlightened system, correspond with the portrait as repeatedly contemplated by the writer of these Remarks. But, although the foundation is laid upon the same facts; yet is it desirable to complete the super-structure by certain modifications of which it appears susceptible, without danger to the general fabric of febrile classification.

vantage is synocha thus closely associated with idiopathic fever; whether intermittent, remittent, or continued?

---

In regard to the West India "*Yellow*" Fever—"Epánetus malignus"—"Typhus icterodes"—"Synochus flavus"—and which was considered by Fordyce a "Semitertian"——the endemic of plethoric new comers from temperate to torrid climates; it should be associated with the synocha plethorica or caúma: *if* it is deemed expedient to detach inflammatory fever—the "general inflammation" of Fordyce, from *idiopathic* fever.

In this case, the term *Fever*, in its application to synocha plethorica and the West India Endemic, must be restrained to its literal import; and not used in its fullest latitude, as including the idea formed from the consideration of the idiopathic disease. For experience proves, that although inflammation greatly *aggravates* the symptoms of *idiopathic* fever, it is, nevertheless, *not* its essential constituent. While the existence of synocha with various topical determination to particular organs, produced by the obvious application of inordinate stimuli, in situations free from the causes of idiopathic fever, equally manifests the difference which subsists between them.

17, Wigmore Street, Cavendish Square,
March 4, 1818.

